# Apomixis: genetic basis and controlling genes

**DOI:** 10.1093/hr/uhac150

**Published:** 2022-07-02

**Authors:** Yuantao Xu, Huihui Jia, Chunming Tan, Xiaomeng Wu, Xiuxin Deng, Qiang Xu

**Affiliations:** Key Laboratory of Horticultural Plant Biology (Ministry of Education), Huazhong Agricultural University, Wuhan, Hubei 430070, China; Key Laboratory of Horticultural Plant Biology (Ministry of Education), Huazhong Agricultural University, Wuhan, Hubei 430070, China; Key Laboratory of Horticultural Plant Biology (Ministry of Education), Huazhong Agricultural University, Wuhan, Hubei 430070, China; Key Laboratory of Horticultural Plant Biology (Ministry of Education), Huazhong Agricultural University, Wuhan, Hubei 430070, China; Key Laboratory of Horticultural Plant Biology (Ministry of Education), Huazhong Agricultural University, Wuhan, Hubei 430070, China; Key Laboratory of Horticultural Plant Biology (Ministry of Education), Huazhong Agricultural University, Wuhan, Hubei 430070, China

## Abstract

Apomixis is the phenomenon of clonal reproduction by seed. As apomixis can produce clonal progeny with exactly the same genotype as the maternal plant, it has an important application in genotype fixation and accelerating agricultural breeding strategies. The introduction of apomixis to major crops would bring many benefits to agriculture, including permanent fixation of superior genotypes and simplifying the procedures of hybrid seed production, as well as purification and rejuvenation of crops propagated vegetatively. Although apomixis naturally occurs in more than 400 plant species, it is rare among the major crops. Currently, with better understanding of apomixis, some achievements have been made in synthetic apomixis. However, due to prevailing limitations, there is still a long way to go to achieve large-scale application of apomixis to crop breeding. Here, we compare the developmental features of apomixis and sexual plant reproduction and review the recent identification of apomixis genes, transposons, epigenetic regulation, and genetic events leading to apomixis. We also summarize the possible strategies and potential genes for engineering apomixis into crop plants.

## Introduction

Generally, angiosperms go through sporophytic and gametophytic generations alternately, and produce future generations by sexual reproduction. However, some plants can also reproduce asexually by apomixis. Apomixis is an asexual reproduction process that produces seeds in the absence of meiosis and fertilization [1, 2]. As apomictic plants can produce clonal offspring that fully retain the genotype of their mother plant through seeds, apomixis can provide many agronomic advantages for crop production: the stable fixation of heterosis through seed; the rapid generation of new superior germplasms; the simplification of hybrid seed production procedures; and the purification and rejuvenation of some vegetatively propagated varieties, such as perennial woody fruit trees [3]. Applying apomixis to the seed production of crops will drive a new green revolution in agricultural science [4].

Apomixis was initially discovered in *Alchornea ilicifolia* [5], and subsequently had been described in more than 400 flower plant species [6]. Many important genera in Asteraceae and Poaceae are reported as typical apomictic plants, such as *Hieracium*, *Taraxacum*, and *Pennisetum*. Some species in these genera are widely studied to dissect the genetic control of apomixis [7–12]. Apomixis also occurs widely in horticultural crops, including citrus [13], crabapple [14], walnut [15], mango [16], pepper [17], and Chinese chive [18]. However, apomixis is relatively infrequent in major crop species [6]. Understanding the mechanism and control of apomixis in existing apomictic plants is the prerequisite for applying apomixis in agriculture.

In this review, the developmental features of apomixis and sexual plant reproduction are described. We summarize the recent understanding of the factors influencing apomixis, including genetic control, transposons, epigenetic regulation, polyploidization, and hybridization. We also propose possible strategies and potential genes to create gametophytic or sporophytic apomixis for application in agriculture.

## Developmental features of apomixis and sexual reproduction

**Figure 1:**
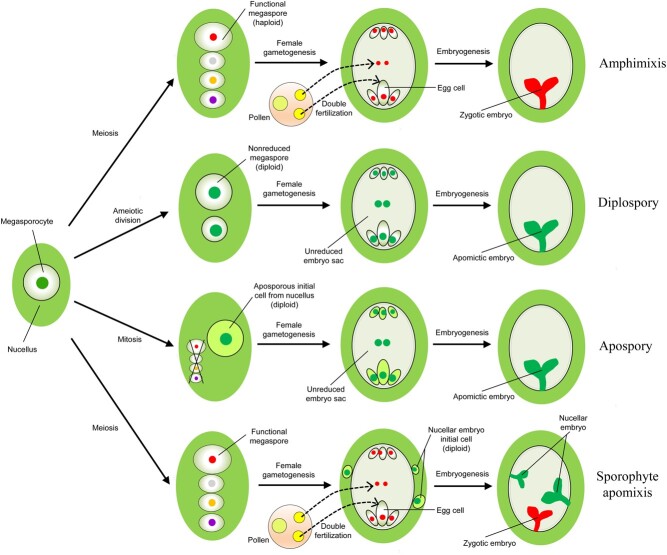
Schematic representation of sexual and apomictic embryo formation. In the process of amphimixis, the megaspore mother cell (MMC) undergoes mitosis and meiosis and develops into a seven-celled, eight-nucleate embryo sac, and then produces a zygotic embryo after double fertilization. For diplospory, the MMC undergoes ameiotic division and divides into two non-reduced megaspores. One of the non-reduced megaspores develops into an unreduced embryo sac. Then the diploid egg cell can directly develop into a parthenogenetic embryo. For apospory, the aposporous initial cell from the nucellus forms an unreduced embryo sac and eventually develops into an asexual embryo. For sporophyte apomixis, at the same time as normal amphimixis, the nucellus-derived nucellar embryo initiation cells divide rapidly and enter the embryo sac, forming one or more nucellar embryos, which can coexist with the zygotic embryo.

During normal sexual reproduction, the megaspore mother cell (MMC) divides into four reduced megaspores through meiosis. Three of these megaspores undergo apoptosis and the remaining functional megaspore develops into a seven-celled, eight-nucleate embryo sac, consisting of one egg cell, one central cell, two synergid cells, and three antipodal cells. When the pollen tube penetrates into the embryo sac, double fertilization occurs [19]. One sperm cell fuses with the egg cell to form a zygote, while the other sperm cell fuses with the central cell and then develops into endosperm, which provides nutrients for embryo development (Fig. 1). In the process of sexual reproduction, meiosis ensures the formation of a reduced embryo sac. Double fertilization not only produces the zygote and triploid nucleus, but also activates the initial development of the zygote by complicated signals from both egg cell and sperm cells [20, 21].

Compared with sexual reproduction, apomixis alters several steps during the initiation and formation of the female germline and produces an asexual embryo with a genotype identical to that of the mother plant (Fig. 1). Based on the origin of the embryo, apomixis can be divided into two types, gametophytic apomixis and sporophytic apomixis (adventitious embryo) [1]. Gametophytic apomixis refers to the asexual embryos derived from the unreduced embryo sac, which can be further divided into diplospory and apospory according to the origin of the cell that initiates unreduced embryo sac formation [22, 23]. In diplospory, the MMC undergoes a modified meiosis and divides into two non-reduced megaspores. One of the unreduced megaspores develops into a diploid embryo sac, in which the diploid egg cell can directly develop into a parthenogenetic embryo without double fertilization [24]. In apospory, a nucellar (somatic) cell near the MMC acquires a gametophytic fate and directly gives rise to the gametophytic lineage without meiosis. The apomictic germline lineage can repress the development of the sexual gametophyte and form an unreduced embryo sac, in which a parthenogenetic embryo is directly developed from the diploid egg cell without fertilization. In some cases, many aposporous initial cells occur in a single ovule and develop into more than one aposporous embryo sac [25, 26]. Gametophytic apomixis completely replaces amphimixis, and is regarded as obligate apomixis [23], while in most apomictic plants both sexual and asexual reproduction processes occur simultaneously in the same ovule, which is termed facultative apomixis [27]. In both diplosporous and aposporous ovules, the endosperm can develop spontaneously without fertilization or through pseudofertilization, providing nutrients for the development of the embryo [19].

For sporophytic apomixis, adventitious embryos are developed from nucellar or integument cells and coexist with the zygotic embryo, leading to the development of a polyembryonic ovule [28]. Generally, the adventitious embryo initial cells appear to be morphologically distinguishable after the formation of the sexual embryo sac. Then they enter the sexual embryo sac and compete with the sexual embryo for nutrients. The survival of the adventitious embryo depends on the fertilization of the sexual embryo sac, which can offer important nutrient and growth signals from the fertilized endosperm [29]. Multiple adventitious embryos can initiate in an individual ovule (Fig. 1).

## Apomixis-controlling loci and related genes

From an evolutionary perspective, apomixis may have evolved from the same molecular framework as that which supports sexual reproduction. When sexual reproduction is aborted as a result of the mutation of corresponding genes, apomixis occurs to overcome infertility. In *Arabidopsis*, a set of mutants have been reported to display phenotypes resembling apomixis (Table 1), such as *ago9* [30] and *swi1* [31], which participate in chromatin remodeling; *spo11-1/2* [32, 33], *mtopVIB* [34], *dfo* [35], *prd1* [36], and *rad50* [37, 38], which are involved in double-strand break formation; *dmc1* [39], *msh4* [40], and asy1 [41], which are essential for chromosome synapsis; *rec8* [42], *scc3* [43], and *ahp2* [44], which are involved in the first meiotic division; *osd1* [45] and *tam* [46, 47], which are related to the meiosis I–meiosis II transition; *tdm1* [48], which controls meiotic termination after meiosis II; *msi1* [49], which is able to initiate parthenogenetic development; *cenh3* [50], which can induce haploid formation; and *fie* [51] and *fis* [52], which can induce endosperm development without fertilization. Most apomictic plants are facultative, which offers the possibility of genetic analyses of apomixis. In all species studied so far apomixis has been proved to be heritable. In citrus and mango, inheritance of sporophytic apomixis as single dominant locus has been proposed [16, 53], while in some diplosporous apomicts genetic loci controlling the key steps of apomixis (apomeiosis, parthenogenesis, and automatic endosperm development) are independent of each other. For example, two separate loci that control diplospory and parthenogenesis have been identified in *Erigeron* and *Taraxacum* species [54, 55]. Apospory and parthenogenesis are determined by two different loci in *Hypericum* [56], *Poa* [57], and *Cenchrus* [58] species. In *Hieracium*, three independent loci, *LOA*, *LOP*, and *AutE*, have been discovered to control apospory, parthenogenesis, and autonomous endosperm development, respectively [59, 60].

**Table 1:** Information on candidate genes related to apomixis

**Component of apomixis**	**Gene**	**Description**	**Genus**	**References**
Apomeiosis	*APOLLO*	*APOLLO* is associated with egg cell formation in apomicts. It is highly expressed in apomictic ovules.	*Boechera*	64, 65
	*UPGRADE2*	*UPGRADE2* represents a long non-coding RNA and its expression is related to the formation of unreduced pollen.	*Boechera*	66
	*AGO104*	*AGO104* is involved in chromatin condensation during meiosis. Mutation of *AGO104* can produce an apomixis-like phenotype, producing functional unreduced female gametes.	*Tripsacum*	67
	*PAIR1*	PAIR1 protein is essential for homologous chromosome pairing in early meiotic prophase in rice.	*Oryza*	68
	*ago9*	*AGO9*-dependent sRNA silencing is important for specification of cell fate and initiation of gametogenesis in the *Arabidopsis* ovule.	*Arabidopsis*	30
	*swi1*	*SWI1* encodes an unknown protein that is important for sister chromatid cohesion in the meiosis process.	*Arabidopsis*	31
	*spo11-1/2*	*SPO11-1* and *SPO11-2* encode Topo VIA proteins, which can induce meiotic double-strand break (DSB), which is required for meiotic recombination.	*Arabidopsis*	32,33
	*mtopVIB*	*MTOPVIB* encodes Topo VIB protein, which can interact with Topo VIA proteins to promote meiotic DSB formation.	*Arabidopsis*	34
	*dfo*	*DFO* is involved in DSB formation. Mutation of *DFO* severely affected homolog synapsis and recombination during meiosis.	*Arabidopsis*	35
	*prd1*	*PRD1* participates in meiotic recombination and is required for meiotic DSB formation.	*Arabidopsis*	36
	*rad50*	Rad50 protein is required for telomere maintenance. Mutation of Rad50 will stimulate chromosomal recombination.	*Arabidopsis*	37,38
	*dmc1*	*DMC1* is involved in meiotic recombination. Mutants of *DMC1* exhibit defects in meiotic DSB formation.	*Arabidopsis*	39
	*msh4*	*MSH4* is involved in crossover formation at the early step of recombination.	*Arabidopsis*	40
	*asy1*	*ASY1* plays an essential role in homologous chromosome synapsis.	*Arabidopsis*	41
	*rec8*	Cohesin Rec8 plays an important role in reductional chromosome segregation.	*Arabidopsis*	42
	*scc3*	SCC3 protein is essential for the maintenance of centromere cohesion.	*Arabidopsis*	43
	*ahp2*	*AHP2* is involved in bivalent formation and homologous chromosome segregation.	*Arabidopsis*	44
	*osd1*	*OSD1* mutants cannot go into the second meiotic division.	*Arabidopsis*	45
	*tam*	*TAM* encodes an A-type cyclin that is involved in both meiosis I and meiosis II.	*Arabidopsis*	46,47
	*tdm1*	*TDM1* is essential for meiotic termination after meiosis II.	*Arabidopsis*	48
Apospory	*QGJ*	*QGJ* is involved in the development of non-reduced embryo sacs in apomictic plants.	*Paspalum*	69
	*GID1*	Ectopic expression of *GID1* leads to the occurrence of MMC-like cells in the nucellus that do not have MMC identity.	*Brachiaria*	70
	*DEFICIENSH*	*DEFICIENSH* may be related to cellular differentiation of the MMC and megagametogenesis.	*Hieracium*	71
	*PnTgs1-like*	*PnTgs1-like* probably determines the fate of nucellar cells, as its reduced expression is associated with initiation of the apomictic pathway.	*Paspalum*	72
	*SERK*	Activation of *SERK* in nucellar cells can induce formation of the aposporous initial cell and development of the asexual embryo sac.	*Poa*	73
Endosperm development	*ORC3*	Defective *ORC3* mutants exhibit a normal female gametophyte but development of the embryo and endosperm is abolished.	*Paspalum*	74
	*FIE*	Expression of *FIE* is negatively correlated with parthenogenesis capacity. Mutant *FIE* allows endosperm development without fertilization.	*Malus, Arabidopsis*	51,75,76
	*fis*	*FIS* controls seed development after double fertilization. In *fis* mutants, partial development of seeds can occur without pollination.	*Arabidopsis*	52
Parthenogenesis	*ASGR-BBML*	*ASGR-BBML* is expressed in unfertilized egg cells of apomictic *Pennisetum squamulatum* and activation of its expression in sexual pearl millet can also trigger parthenogenesis.	*Pennisetum*, *Cenchrus*	12,77
	*PAR*	The dominant *PAR* allele of dandelion is specifically expressed in egg cells and can trigger embryogenesis without fertilization.	*Taraxacum*	78
	*MTL1*	*MTL1* encodes a pollen-specific phospholipase that is involved in fertilization. Mutation of the *MTL1* gene can induce haploid formation in maize.	*Zea*	79
	*msi1*	The *MSI1* gene functions in chromatin assembly. Mutants of *MSI1* can produce parthenogenetic embryos.	*Arabidopsis*	49
	*cenh3*	Alteration of centromere-specific histone CENH3 can induce genome elimination and haploid formation.	*Arabidopsis*	50
Adventitious embryogenesis	*RWP*	The *CitRWP* gene co-segregates with the citrus nucellar embryo and is preferentially expressed in nucellar embryo initiation cells. Loss of *CitRWP* function can abolish nucellar embryogenesis in citrus.	*Citrus*	80,81
	*AGL11*	*AGL11* is a MADS-box transcription factor and is preferentially expressed at the apomictic nucellar embryo stage in *Zanthoxylum bungeanum*. Ectopic expression of *ZbAGL11* can lead to abnormal flower development and induce apomixis-like phenotypes in *Arabidopsis*.	*Zanthoxylum*	82

Despite the discovery of multiple apomixis-linked loci in various species, it is still difficult to identify the specific genes controlling apomixis, as the apomixis-linked loci are usually recombination-inhibited and located in repetitive regions [61–63]. So far, a few genes have been identified that are involved in different components of apomixis (Table 1). For apomeiosis, two different candidate genes, *APOLLO* (apomixis-linked locus) and *UPGRADE2* (unreduced pollen grain development), have been identified in *Boechera*. The expression of *APOLLO* and *UPGRADE2* is strongly correlated with the formation of apomeiotic eggs and pollen, respectively [64–66]. In *Tripsacum*, *AGO104*, which is involved in DNA methylation, is proposed to be required for proper chromatin condensation during meiosis [67]. In *Oryza sativa*, the *PAIR1* gene was identified to play an essential role in chromosome synapsis in early meiotic prophase [68]. For apospory, a MAP3K-coding *QUI-GONJINN* (*QGJ*) gene in *Paspalum notatum* is suggested to be essential for aposporous embryo sac formation [69]. In *Brachiaria brizantha*, the specific expression pattern of *GIBBERELLIN-INSENSITIVE DWARF1* (*GID1*) suggests its function in aposporous initial cell differentiation to form the aposporic embryo sac [70]. In apomictic *Hieracium*, transient downregulation of a floral organ-identity gene (*DEFICIENS*) in the chalazal region is associated with aposporous initial cell formation [71]. Similarly, in *P. notatum*, *PnTgs1-like* was proposed to play an important role in nucellar cell fate, as its reduced expression is associated with the initiation of the aposporous pathway [72]. In *Poa pratensis*, *PpSERK* is proposed to be responsible for the formation of the aposporous initial cell and the development of the asexual embryo sac [73]. For autonomous endosperm formation, *ORC3* and *FIE* were proved to be vital candidate genes. The accurate expression of *ORC3* in germ cell lineages determines the development of the endosperm in apomictic *Paspalum simplex* [74]. In *Malus hupehensis*, *FIE* is involved in the regulation of asexual seed formation [75]. Ectopic expression of *MhFIE* in tomato produces parthenocarpic fruit [76]. For parthenogenesis, *ASGR-BBML* has been proved to be the most promising candidate. *ASGR-BBML* is expressed in unfertilized egg cells of apomictic *Pennisetum squamulatum* and transformation of sexual pearl millet with the *ASGR-BBML* gene can trigger parthenogenesis [12, 77]. Recently, a *PARTHENOGENESIS* (*PAR*) gene was isolated from apomictic common dandelion, which can induce embryo-like structures without fertilization in lettuce [78]. In addition, mutation of a pollen-specific phospholipase, MTL1, can induce paternal genome elimination and haploid formation in maize and rice [79]. For adventitious embryogenesis, several candidate genes have also been reported. In citrus, the *CitRWP* gene was identified by genetic analysis of segregating populations and proved to be associated with nucellar embryo formation [80, 81]. In another typical sporophytic apomictic plant, *Zanthoxylum bungeanum*, the expression of *AGL11* shows correlation with nucellar embryo development and its ectopic expression can lead to abnormal flower development and simulate apomixis phenotypes in *Arabidopsis* [82].

## Miniature inverted-repeat transposable element transposons mediate activation of apomixis-controlling genes

**Figure 2:**
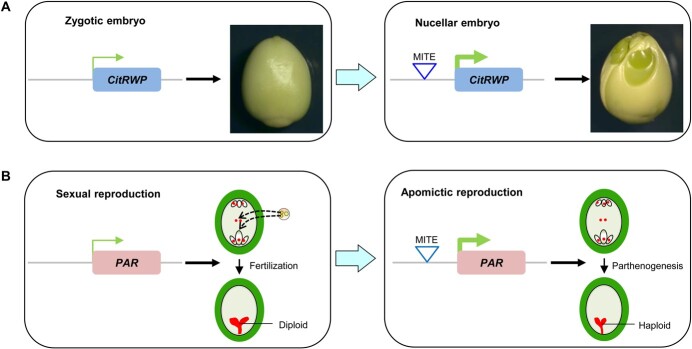
Typical cases of apomixis induced by MITE transposons. (A) A MITE transposon inserted in the *CitRWP* promoter activates gene expression, leading to multiple nucellar embryos in one seed of the polyembryonic citrus. In monoembryonic citrus there are no MITE transposon inserts in the *CitRWP* promoter and the gene is weakly expressed. (B) In sexual dandelions the *PAR* allele from the female parent with no MITE transposon insertion is not expressed in the egg cell, and the sexual diploid embryo comes from double fertilization. In polyploid apomicts the *PAR* allele with a MITE transposon insertion in its promoter is activated in the egg cell. Then the egg cell can directly develop into a haploid embryo without fertilization; this is parthenogenesis.

Transposon insertions can affect the expression and function of adjacent genes and can cause phenotypic changes in plants [83–85]. With the development of research on apomixis, some evidence suggests that transposons may be involved in apomixis. In both aposporous *Cenchrus ciliaris* and *P. squamulatum*, the apospory-specific genomic region (*ASGR*) is located on a single chromosome that contains transposons and repeated sequences [9, 86, 87]. The hemizygous chromosomal region containing the *LOSS OF APOMEIOSIS* (*LOA*) locus in *Hieracium* also has abundant complex repeats and transposon sequences [88]. These structural features of the apomixis loci suggest that transposons might take part the induction or maintenance of apospory in these plants. Notably, our previous genetic analysis identified a miniature inverted-repeat transposable element (MITE) transposon insertion in the promoter region of the candidate gene (*CitRWP*) controlling sporophytic apomixis in *Citrus* [80]. This MITE transposon showed complete co-segregation with the polyembryony trait of *Citrus* in both natural and segregating populations. In polyembryonic citrus varieties, the *CitRWP* gene with a MITE transposon insertion is highly expressed. While in monoembryonic varieties, no MITE transposon insertion was found in the promoter region of the *CitRWP* gene and its expression was barely detectable. All these results suggest that the MITE transposon insertion in the promoter region of the *CitRWP* gene is required to enable sporophytic apomixis in citrus (Fig. 2A). Similarly, in apomictic dandelion (*Taraxacum officinale*) and hawkweed (*Hieracium piloselloides*) MITE transposons also exist in the upstream region of the parthenogenesis gene (*PAR*) [78]. The MITE-containing promoter from dandelion can activate the *PAR*-homologous gene from sexual lettuce to reproduce the dandelion parthenogenetic phenotype, suggesting the decisive effect of the MITE transposon on parthenogenesis (Fig. 2B).

In both *Citrus* and dandelion, the MITE transposons inserted in the promoter region may be associated with upregulation of the adjacent genes, thereby controlling apomixis. It is likely that MITE insertions in the promoter of *CitRWP* or *PAR* genes lead to a transition from sexual reproduction to adventitious or parthenogenetic embryo development. Generally, MITE insertion in the promoter may impact gene expression through two different mechanisms: (i) by introducing a spatiotemporally specific activating element within the MITE; and (ii) by disrupting a repressive regulatory element that normally represses adjacent gene expression. Another mechanism influencing alteration of DNA methylation patterns should also be considered. Recently, a DNA methylome analysis revealed hypermethylation in the promoter of *CitRWP* in polyembryonic citrus, which contains a MITE insertion, while hypomethylation was detected in the promoter of *CitRWP* in monoembryonic citrus without a MITE insertion [89]. This result suggests that the MITE insertion may be related to the hypermethylation of *CitRWP*, which might activate gene transcription and further enable cells in the ovules of polyembryonic citrus to switch to an apomictic pathway.

## Epigenetic regulation of apomixis

The initiation of apomixis is believed to be attributable to the downregulation of important genes in sexual reproduction, and epigenetic regulation enables reversible conversion between the two reproductive modes in plants. Transcriptome comparison of apomictic *Boechera*, *Hieracium*, and *Hypericum* with related sexual lines revealed changes in siRNA synthesis and RNA-directed DNA methylation (RdDM)-related gene expression [90–92]. DNA methylation analysis showed that the overall methylation level of gametophyte-apomictic *C. ciliaris* [93] and sporophyte-apomictic citrus [89] was lower than that of sexually reproducing plants, suggesting that hypomethylation is related to apomictic reproduction, while in the gametophyte-apomictic *Paspalum* treatment with the DNA methylation inhibitor 5-azacytidine significantly reduced parthenogenesis frequency, suggesting that a high DNA methylation level may maintain apomictic reproduction [94].

The absence of important epigenetic pathway genes in model plants can lead to a phenotype resembling apomixis. Multiple mutants involved in siRNA synthesis and the RdDM pathway can form additional gametophytic cells, similar to what occurs in diplospory, such as *ago9*, *sgs3*, *rdr2*, *rdr6*, and *dcl3* [95]. Recent studies have shown that *AGO9* and *RDR6* gene mutations lead to ectopic expression of *SPL*/*NZZ*, which controls the differentiation of MMC cells, resulting in multiple MMC-like cells in the ovule [96].

## Genetic events lead to apomixis: polyploidization and hybridization

Hybridization and polyploidization can widely activate transposable elements that are silenced by epigenetic modifications [83, 97]. Most gametophytic apomicts are polyploid, and a causal relationship has been proposed between apomixis and polyploidization [6]. With respect to the cytological mechanism of apomixis, there is really a link between apomixis and polyploidization, as synthetic induction of polyploidy can induce apomixis from sexual plants [98, 99]. Nevertheless, apomixis can also occur in diploid plants [100, 101], suggesting that polyploidy is not a prerequisite of apomixis. The causal relationship between apomixis and polyploidization remains unclear. The point of view has been put forward that a polyploid genome can promote the optimum expression of apomixis [99], while it is also proposed that polyploidization may be a result of apomixis, which confers genomic stability. During the apomixis process, apomeiosis and parthenogenesis may increase the frequency of polyploidization [102, 103].

Most apomictic polyploids are allopolyploids, formed by hybridization between genetically divergent diploid species [104]. So it is also speculated that hybridization, rather than polyploidy, leads to apomixis [27, 105]. In genome-duplicated hybrids, asynchronous expression of the two sets of genes involved in female reproduction may result in precocious embryo sac initiation and embryogenesis [6]. In *Boechera*, diploid apomicts show high heterozygosity caused by the conjunction of disparate genomes, which suggests that the genomic consequences of hybridization may be related to gametophytic apomixis in this genus [106]. A hybrid origin of apomixis had also been proposed in the *Ranunculus cassubicus* complex, and some unique alleles that resulted from genomic reorganization in allopolyploids might trigger apomixis [107]. Additionally, some sporophytic apomictic species, such as *Citrus* [108], *Mangifera* [109], and *Zanthoxylum* [110], all have a high level of heterozygosity.

## Perspectives for future study and applications of apomixis in breeding

Apomixis is a fascinating phenomenon with great application potential for agriculture. Although increasing numbers of genes associated with apomixis have been identified, the gene regulatory network and molecular mechanisms of apomixis are not clear. Based on the candidate genes currently identified by genetic analysis, further studies are needed to dissect the upstream and downstream regulators. And a clear regulatory network of each component of apomixis will provide a strong foundation for the engineering of apomixis in crops. As apomixis occurs randomly in some genera of angiosperms and all steps of the apomixis process have evolved several times independently, exploring the origin and evolution of apomixis may provide more clues about the mechanism of apomixis. For example, the *CitRWP* gene has been proved to control nucellar embryony in *Citrus*, but not in its related genera in Rutaceae, including *Zanthoxylum*, *Murraya*, and *Poncirus*, although these genera exhibit a form of sporophytic apomixis similar to that of *Citrus* [111]. It is probable that all the genera in Rutaceae undergo the same apomixis pathway, but the mutations associated with apomixis in different genera may have occurred at different nodes of the pathway. Thus, identification of the different mutations leading to nucellar embryogenesis in each genus may contribute to deeper understanding of the regulatory pathways of sporophytic apomixis in Rutaceae.

Artificial creation of apomixis in crops is an effective way to fix heterosis and the ultimate goal of studying apomictic reproductive traits. Several studies have reported the engineering of gametophytic apomixis. The *MiMe* (substitute mitosis for meiosis) system was first created in *Arabidopsis* by simultaneous mutation of three key meiotic genes (*SPO11-1*, *REC8*, and *OSD1*) [112]. Hybridization of a *cenh3* null mutant expressing altered CENH3 protein, which can induce centromere-mediated genome elimination, with *MiMe* plants produced clonal reproduction though seeds [113]. In rice, triple mutations of three key meiotic genes (*PAIR1*, *REC8*, and *OSD1*) can also turn mitosis to meiosis (*MiMe*) [114]. Multiplex editing of the three key meiotic genes and the *MTL* gene resulted in plants that can propagate clonally through seeds [115]. Moreover, *MiMe* combined with the expression of a parthenogenesis gene, *BBM1*, in the egg cell can also induce clonal progeny of hybrid rice that retain genome-wide parental heterozygosity [116]. However, there are still some limitations in the application of the above synthetic apomixis strategies. The *MiMe* system combined with *CENH3* system depends on hybrid pollination [113], which restricted the commercial production of clonal seeds. In both the *MiMe* combined with *MTL1* system and the *MiMe* combined with *BBM1* system, the clonal seeds exhibited relatively low fertility, possibly due to the low frequency of parthenogenesis [115, 116]. Current apomixis strategies might be improved by increasing parthenogenesis induction efficiency. In addition, the *MiMe* combined with *BBM1* system requires self-pollination to initiate endosperm development and thus sexual seeds are also produced together with the clonal seeds. This system could potentially be improved by integrating genes that can promote autonomous endosperm development, such as *ORC* [74] and *FIE* [51].

As the mechanisms of sporophytic apomixis are less studied, application of sporophytic apomixis has been difficult. The *CitRWP* gene identified in citrus is a potential candidate to create sporophytic apomictic crops [80]. RWP-RK domain-containing (RKD) genes play important roles in the maintenance of egg-cell identity and their ectopic expression can promote somatic embryogenesis in *Arabidopsis* [117]. The specific expression of *CitRWP* in citrus ovules may enable the nucellar cells to acquire an embryonic fate. Additionally, a C2H2 zinc-finger domain-containing transcription factor gene (*CitZFP*), which is homologous to the dandelion parthenogenesis gene (*PAR*), is specifically expressed in apomictic cells [89]. This gene may be another candidate gene for engineering sporophytic apomixis. Further studies on the function and regulation of *CitRWP* and *CitZFP* genes in citrus are necessary for the utilization of sporophytic apomixis in apomixis breeding.

## Acknowledgements

We thank Prakash Babu Adhikari for critical reading of the manuscript. This study was financially supported by the National Natural Science Foundation of China (32001997), Major Special Projects and Key R&D Projects in Yunnan Province (202102AE090054), and the National Postdoctoral Program for Innovative Talents (BX20200146).

## Author contributions

Q.X. and Y.X. planned the outline of the review. Y.X. completed the first draft of the paper. H.J. and C.T. helped with literature collection and discussion. Q.X., X.W., and X.D. revised the paper. All authors approved the final paper.

## Conflict of interest

The authors declare no competing interests.
